# Estimating the discretization dependent accuracy of perfusion in coupled capillary flow measurements

**DOI:** 10.1371/journal.pone.0200521

**Published:** 2018-07-20

**Authors:** Erik A. Hanson, Constantin Sandmann, Alexander Malyshev, Arvid Lundervold, Jan Modersitzki, Erlend Hodneland

**Affiliations:** 1 Department of Mathematics, University of Bergen, Bergen, Norway; 2 Institute of Mathematics and Image Computing, University of Lübeck, Lübeck, Germany; 3 Department of Biomedicine, University of Bergen, Bergen, Norway; 4 Department of Radiology, Haukeland University Hospital, Bergen, Norway; 5 Christian Michelsen Research AS, Bergen, Norway; University of Massachusetts Medical School, UNITED STATES

## Abstract

One-compartment models are widely used to quantify hemodynamic parameters such as perfusion, blood volume and mean transit time. These parameters are routinely used for clinical diagnosis and monitoring of disease development and are thus of high relevance. However, it is known that common estimation techniques are discretization dependent and values can be erroneous. In this paper we present a new model that enables systematic quantification of discretization errors. Specifically, we introduce a continuous flow model for tracer propagation within the capillary tissue, used to evaluate state-of-the-art one-compartment models. We demonstrate that one-compartment models are capable of recovering perfusion accurately when applied to only one compartment, i.e. the whole region of interest. However, substantial overestimation of perfusion occurs when applied to fractions of a compartment. We further provide values of the estimated overestimation for various discretization levels, and also show that overestimation can be observed in real-life applications. Common practice of using compartment models for fractions of tissue violates model assumptions and careful interpretation is needed when using the computed values for diagnosis and treatment planning.

## 1 Introduction

Quantitative measurements of hemodynamic medical parameters based on tracer kinetic modeling are widespread both in research and in clinical practice [1–3]. Perfusion maps, as well as other parameter maps arising from tracer kinetic modeling can be combined with anatomical information and have proven to be of particular value in e.g. stroke studies or localization of trauma. Among the physiological parameters obtainable from tracer kinetic modeling, perfusion has been found particularly difficult to describe reliably on a voxel-basis [4]. These limitations are caused by issues in the numerical implementation [4], but might also depend on over-simplified dynamic models, which were originally designed to describe larger volumes of interest [5]. Perfusion is a term describing flow in highly complex microcirculatory networks, and a reliable representation of perfusion is problematic due to imprecise mathematical definitions of the concept.

Uncertainties in signal-to-concentration convertion are modality dependent but are considered to be outside the scope of the current work. For the remaining, we will assume that a tracer concentration field is obtainable from the imaging data and that modality-dependent characteristics like signal decay are possible to handle within the model framework.

In the current work we focus on the fundamental problem of perfusion as a discretization dependent parameter as measured within traditional 1-compartment models and how it scales with voxel size. This problem was previously identified by several authors [6–8] but has not been sufficiently well addressed in clinical studies on perfusion, nor has it been described in depth mathematically. A drawback in previous studies evaluating the performance of 1-compartment models has been that synthetic ground truth data and perfusion estimations are performed with similar models, thus not challenging the validity of the model itself, but only studying issues related to numerical stability and noise resistance. In the current work we circumvent this problem by generating synthetic flow data using a continuous flow field model substantially different from the 1-compartment models essentially disconnected in space. Despite the scale dependency of perfusion estimates, it is widely accepted that perfusion estimates can provide valuable information about pathological conditions. However, it is not known to what extent the discretization dependent error is homogeneously distributed, how it scales with voxel size, or whether it depends on local geometry, capillary density, anisotropy or other conditions affecting delivery of arterial blood to the capillary system. In the current work we rigorously attack several of these challenges.

Traditional one-compartment (1C) models like deconvolution or the maximum slope model are able to recover perfusion accurately if applied to the entire domain fed by the incoming flow. However, when applying the traditional models to isolated parts of the full system we are able to confirm that local perfusion in coupled systems is indeed discretization dependent and not physically correct to be used as a measure of arterial blood delivery. In order to highlight this issue, two ground truth values of voxelwise perfusion are presented: A definition *P*_v_ describing the local inflow into a voxel, but otherwise not fulfilling the traditional ideas of perfusion, and then a tailored definition of perfusion *P*_s_ for continuous models.

Using these definitions on perfusion, the main aim of our work is to quantify the discrepancy between a numerical ground truth and perfusion as measured using traditional 1C models. A thorough quantification of the error is valuable information for a critical interpretation of obtained perfusion values in clinical studies. In particular, the results of our work are useful within multi-centre studies on perfusion. These studies are particularly susceptible to various discretizations arising from the usage of different hardware, acquisition protocols and post-processing tools, and where the interpretation and comparison of absolute perfusion values therefore should be undertaken with particular care.

## 2 Materials and methods

### 2.1 Traditional one-compartment models

Two widely used 1C pharmacokinetic models for measurements of CBF and CBV are the deconvolution- and the maximum slope model [2–4]. For the remaining, they are referred to as *traditional* models. Let Ω_*i*_ be an arbitrary control volume with one inlet and one outlet, and let *C*(*t*) denote the average contrast agent (CA) concentration within Ω_*i*_ at timepoint *t*. The traditional models assume that the change of concentration at timepoint *t* can be described by the ordinary differential equation

$$\begin{array}{c} C^{\prime }\left(t\right)=P_{\text{a}}c_{\text{a}}\left(t\right)-P_{\text{v}}c_{\text{v}}\left(t\right),\;C\left(0\right)=0. \end{array}$$ (1)

Here, *c*_a_, *c*_v_ are the plasma CA concentrations at the inlet and outlet of Ω_*i*_ and *P*_a_, *P*_v_ is the normalized flow [mm^3^s^−1^mm^−3^] at these locations. In the following, it is assumed that the plasma tracer concentration *c*_a_ at the inlet is known. In clinical practice this can be accounted for by measuring *c*_a_ in a feeding artery [9]. Since *c*_v_ is usually unknown, additional assumptions need to be made if one wants to reconstruct the perfusion *P*_a_ from a given tissue curve *C*. The convolution model and the maximum slope (MS) model diverge in further assumptions.

#### 2.1.1 The convolution model

For derivation of the deconvolution model, one approach is to assume there is an unknown probability distribution of transit times *h* through Ω_*i*_, cf. [1]. This leads to

$$\begin{array}{c} P_{\text{v}}c_{\text{v}}\left(t\right)=P_{\text{a}}\left(h*c_{\text{a}}\right)\left(t\right):=P_{\text{a}}\int_{0}^{t}c_{\text{a}}\left(s\right)h\left(t-s\right)ds. \end{array}$$ (2)

Combining this with (1) yields *C*′(*t*) = *P*_a_*c*_a_(*t*) − *P*_a_(*h* ∗ *c*_a_)(*t*). Integrating and using basic properties of the convolution one obtains the general solution

$$\begin{array}{c} C\left(t\right)=\left(I*c_{\text{a}}\right)\left(t\right) \end{array}$$ (3)

where the *impulse response function* *I* is defined as $I\left(t\right):=P_{\text{a}}\left(1-\int_{0}^{t}h\left(s\right)ds\right)$. The task of identifying *I*(*t*) given a tissue curve *C*(*t*) and an arterial input function *c*_a_(*t*) is a deconvolution problem. If *I*(*t*) is recovered, *P*_a_ can subsequently be estimated as *P*_a_ = max_*t*_*I*(*t*). There are several methods to perform the deconvolution. A standard approach using Fourier-based algorithms is sensitive to the presence of noise [9]. Another class of deconvolution algorithms gaining increasing attention are based on Bayesian modeling [10]. However the numerical handling is still difficult since complex and error-prone numerical integration has to be performed [10]. A popular class among deconvolution algorithms is based on singular value decomposition (SVD) [9]. These algorithms have shown to be robust for a reasonable noise level. Also, they can be easily adapted to be robust against delays in tracer arrival using block-circular structures (bSVD cf. [11]). In order to identify the impulse response function *I*(*t*) from applied data, we hence decided to use the bSVD model as proposed in [11].

#### 2.1.2 The maximum slope model

In the MS model it is assumed that when *c*_a_ has its maximum, only a negligible amount of CA is leaving the system [12]. For this time interval, (1) reduces to

$$\begin{array}{c} C^{\prime }\left(t\right)=P_{\text{a}}c_{\text{a}}\left(t\right),\;C\left(0\right)=0. \end{array}$$ (4)

One can see that if *c*_a_ has a maximum, also *C*′ must have a maximum since stationarity in *P*_a_ is assumed. Hence, it holds that

$$\begin{array}{c} P_{\text{a}}=\frac{\max_{t}C^{\prime }\left(t\right)}{\max_{t}c_{\text{a}}\left(t\right)}. \end{array}$$ (5)

### 2.2 A synthetic model for capillary flow

The validity of traditional 1C models relies on a control volume with only one inlet and one outlet, and that the control volumes are not feeding each other. These assumptions may be violated when we locally describe CA propagation through a larger volume. For this type of model system we instead expect a set of coupled equations where each voxel can be regarded as an inlet for surrounding voxels.

Hence, in order to make a realistic synthetic model for capillary flow, we decided to describe the CA propagation as a spatially coupled transport process, i.e. using partial differential equations (PDE) for transport. This PDE model is used for validation of the traditional models.

A major difference between our coupled flow model and traditional tracer kinetic modeling is the normalization of the flow field. To avoid a discretization dependent flow field for the PDE model, we instead of perfusion use vector valued surface fluid flux **q** = **q**(**x**) [mm^3^s^−1^mm^−2^] as the fluid carrying quantity, in agreement with geoscience and porous media simulation theory. The fluid flux is a vector field describing the volume of fluid per unit time flowing across a sliced unit area of the sample. A detailed outline of how the flow field was obtained can be found in Section 2.7.

### 2.3 A model for indicator dilution

This section describes a model for CA propagation in the tissue. We assume that homogeneously dissolved CA is entering the domain along with the fluid flowing into the ROI via the source, and similarly extracted at a sink. In order to define meaningful and continuous contrast agent concentrations, we first describe CA concentration in an (arbitrarily) small tissue volume Ω_*ε*_. Let *V*_*ε*_ be the volume of Ω_*ε*_ centered around **x** and let *v*_*ε*_ be the blood volume within the same control region. Letting the control region go towards zero volume, the porosity *ϕ*(**x**) ≔ lim_*ε*→0_*v*_*ε*_/*V*_*ε*_ [mm^3^mm^−3^] reflects the local, relative volume of the vascular system. The simplification of porosity as a continuous function is frequently performed in flow simulations. The flux **q**(**x**) as well as the porosity *ϕ*(**x**) are assumed to be stationary and hence independent of time. We further introduce *C* = *C*(**x**, *t*) and *c* = *c*(**x**, *t*) as the average CA concentration within *V*_*ε*_ and *v*_*ε*_, respectively. By definition, we obtain the relation *C*(**x**, *t*) = *ϕ*(**x**)*c*(**x**, *t*). The rate of change of tracer molecules within a control volume Ω_*i*_ can hence be phrased as

$$\begin{array}{c} \frac{d}{dt}\int_{\Omega_{i}}Cdx=\int_{\Omega_{i}}\frac{d}{dt}\left(\phi c\right)dx=\int_{\Omega_{i}}\phi \frac{dc}{dt}dx, \end{array}$$ (6)

where the assumption of stationary *ϕ*(**x**) was used. Assuming mainly transport and marginal diffusion, the change in tracer mass within Ω_*i*_ occurs from advective flow and the source and sink field *Q*(**x**). Let us write the source- and the sink term as *Q*(**x**) = *Q*_si_(**x**) + *Q*_so_(**x**) where *Q*_si_(**x**) < 0 is the sink and *Q*_so_(**x**) > 0 is the source, and zero elsewhere. Note that ∫_Ω_*Q* **d** **x** = 0. Using (6) and following the principle of conservation of tracer molecules, the rate of change of contrast agent in a control volume Ω_*i*_ is modelled as

$$\begin{array}{c} \int_{\Omega_{i}}\phi \frac{dc}{dt}dx+\int_{\partial \Omega_{i}}c\left(q\cdot n\right)dA=\int_{\Omega_{i}}\left(c_{\text{a}}Q_{so}dx+cQ_{si}\right)dx. \end{array}$$ (7)

where **n**(**x**) is the outward unit normal on ∂Ω_*i*_. Eq (7) is consistent with the continuity equation on local form

$$\begin{array}{c} |\begin{array}{cc} \phi \frac{\partial c}{\partial t}+\nabla \cdot \left(cq\right)=c_{\text{a}}Q_{\text{so}}+cQ_{\text{si}} & x\in \Omega ,\;t>0,\\ c(x,t)=0 & x\in \Omega ,\;t=0. \end{array}| \end{array}$$ (8)

for the initial condition *c*(**x**, 0) = 0 in accordance with no initial tracer at *t* = 0. Eq (8) is a linear transport equation in *c*(**x**, *t*). Following [13], (8) admits a unique local solution.

### 2.4 Relating the transport equation model with the traditional deconvolution model for perfusion

In this section we describe how the continuous model is related to the traditional deconvolution model. We will show that in the continuous model the flow into each voxel can be described as a traditional model with arterial input determined by adjacent upstream voxels.

Let us start by modeling the CA concentration in a given voxel Ω_*i*_ using traditional models. For sake of simplicity we assume that *Q*_so_ = *Q*_si_ = 0 within that voxel. It is possible to extend the following approach also to voxels where this is not the case. Define the outward normal vector **n** and voxel face areas of inflow and outflow over the boundary as *S*_in_ ≔ {**x** ∈ ∂Ω_*i*_: **q** ⋅ **n** < 0} and *S*_out_ ≔ {**x** ∈ ∂Ω_*i*_: **q** ⋅ **n** > 0} respectively. For the domain Ω_*i*_ we define the arterial input *c*_in_ as the weighted average of the tracer flux across *S*_in_

$$\begin{array}{c} c_{\text{in}}\left(t\right):=\frac{\int_{S_{\text{in}}}c\left(q\cdot n\right)dA}{\int_{S_{\text{in}}}q\cdot ndA}. \end{array}$$ (9)

We define local perfusion *P*_v_ within Ω_*i*_ as the total feeding fluid inflow divided by the volume,

$$\begin{array}{c} P_{\text{v}}:=-\frac{1}{|\Omega_{i}|}\int_{S_{\text{in}}}q\cdot ndA. \end{array}$$ (10)

Given incompressible flow, the rate of fluid entering the region is the same as the rate of fluid leaving it. Further, let *c*_*i*_(*t*) denote the average fluid CA concentration within Ω_*i*_. Then it holds that *P*_v_ = *P*_out_ and we can describe *c*_*i*_ by the traditional model (1)

$$\begin{array}{c} {\left(\phi c_{i}\right)}^{\prime }\left(t\right)=P_{\text{v}}\left(c_{\text{in}}\left(t\right)-c_{\text{i}}\left(t\right)\right) \end{array}$$ (11)

with solution

$$\begin{array}{c} C_{i}\left(t\right)=\phi_{i}\left(J_{i}*c_{\text{in}}\right)\left(t\right)\;\text{where}\;J_{i}\left(t\right)=\left(P_{\text{v}}/\phi_{i}\right)e^{-(P_{\text{v}}/\phi_{i})t}. \end{array}$$ (12)

**Fig 1:**
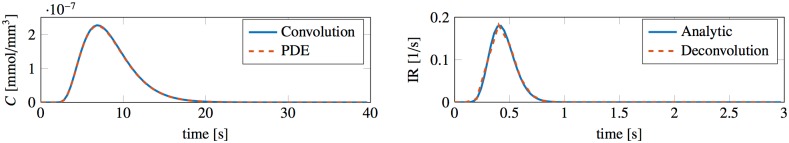
Upstream dependency within traditional models. Left: Red curve shows the tissue concentrations (*C*) of the continuous PDE model at location [32, 35]. Blue curve shows recursive convolution by (12) with experimental value of *P*_v_ = 5328 ml/min/100ml at the given location and *c*_in_ taken locally from upstream voxels around the simulated voxel. The two curves have an almost perfect overlap. Note that the numbers used for the perfusion is unrealisticely high since normalization is performed with respect to the volume of only one voxel. Right: Red curve shows the computed impulse response functions (IR) at location [1, 20] using the global arterial input function. Blue curve shows the analytic impulse response function given by a convolution over all upstream flow. The two curves have an almost perfect overlap. These numerical experiments support that the computed impulse response function by traditional methods is not the directly feeding impulse response function, but rather a recursive impulse response function depending on all upstream voxels.

The arterial input *c*_in_ is determined by (9), which recursively depends on all upstream voxels until the global arterial input is reached. To verify this relationship numerically, we simulated a tissue curve *C*_*i*_(*t*) using both the continuous PDE model as well as analytical recursive convolution by (12). We refer to the latter approach as *local convolution*. The two curves have an almost perfect match, as seen in Fig 1 (left).

It follows by recursion that the concentration at voxel *i* can be written as a convolution of the (global) arterial input function with a weighted average of all upstream impulse response functions. Deconvolving a tissue concentration *C*_*i*_ with the global AIF will yield an impulse response function which depends not only on the local flow and porosity, but on flow and porosity of all upstream voxels. This relationship was also confirmed experimentally: Fig 1 (right) shows the impulse response function determined by analytical recursive convolution and the numerically achieved impulse response function obtained from deconvolving a tissue curve of the continuous model with the global arterial input function. The simulation was performed at location (1, 20) of the digital phantom. The two curves coincide almost perfectly, highlighting the validity of the established theory.

These results show that the PDE model and the convolution model are equal in terms of local, voxelwise flow estimates if the convolution model is applied with the local arterial input. Also, the impulse response function obtained by convolution of the global arterial input function is identical to an analytical recursive convolution along all upstream voxels. This demonstrates that the perfusion recovered by traditional models depends on all upstream flow. However, for meaningful interpretation of the perfusion the entire streamline length within the capillary system needs to be taken into consideration. These results demonstrate that perfusion is depending on the geometry of the streamlines, hence the geometry of the capillary system.

### 2.5 Relating flux with perfusion

The flow model described in (21) uniquely determines the flux field **q**(**x**). However, in pharmacokinetic modeling the parameter of interest is usually CBF, which we will denote by *P*(**x**) as the voxelwise field of perfusion. Surface flux and perfusion are physically distinct, and there are at least two differences between **q**(**x**) and *P*(**x**). First, flux is a vector field and perfusion is a scalar field. Second, the flux is normalized to a surface area and the perfusion is normalized to a volume. Hence the flux describes flow over a surface separating spatial regions, while the perfusion describes blood leaving/entering a compartment within a given volume. According to the common understanding of perfusion, *P*(**x**) is the amount of blood feeding a tissue volume per unit time, with units [mm^3^s^−1^mm^−3^]. In this work we address the fine scale setting, where the perfusion is taking place on a voxel level. At this level, a clearer understanding of how perfusion relates to the flux is desirable.

One straightforward approach for converting flux into perfusion could be to estimate the perfusion as the total inflow (or outflow) of fluid (e.g. arterial blood) into a control region per unit time, and then normalizing with the control region volume. This is a valid approach only if the control regions are not feeding each other, and is therefore well-founded for the entire organ, in line with the theoretical foundation of traditional compartment models for perfusion where a control region has its own source of feeding arterial blood, independent of neighbour regions.

**Fig 2:**
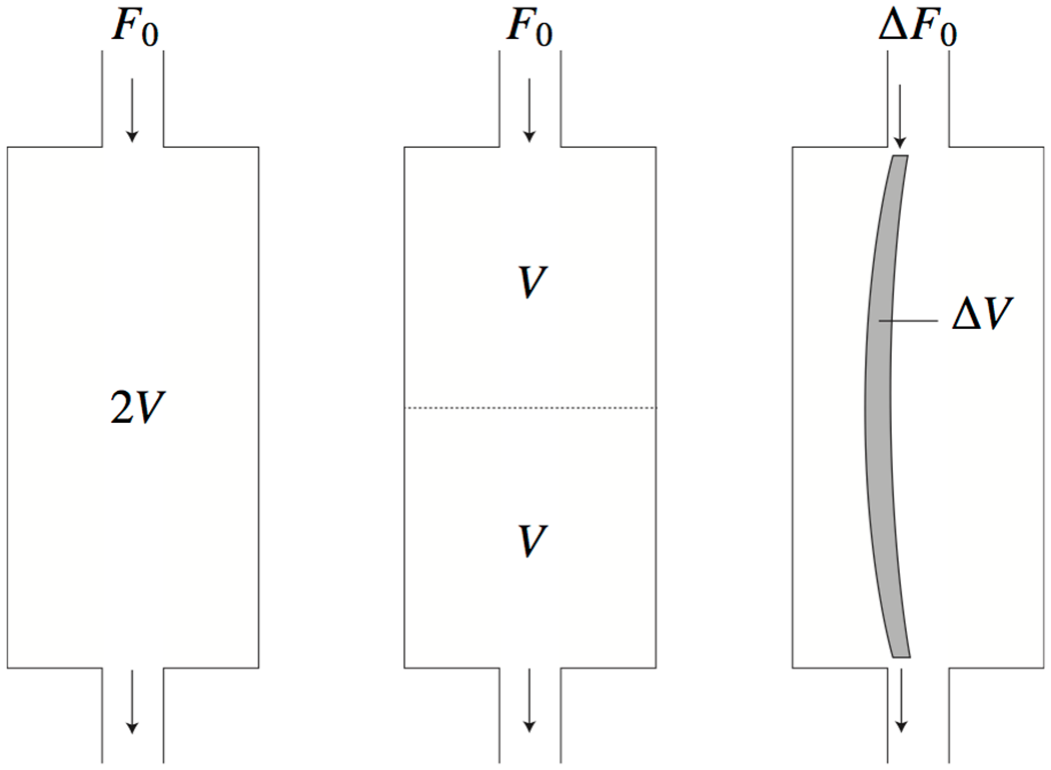
Perfusion as a discretization dependent measure. Perfusion within a small volume. Left: A compartment with volume 2*V* is exposed to a flow *F*_0_ [mm^3^s^−1^] of fluid. By definition, the perfusion within this compartment becomes *P*_1_ = *F*_0_/(2*V*). Middle: The same volume is divided into two compartments (e.g. voxels), and the perfusion for each of the compartments becomes *P*_2_ = *F*_0_/*V* = 2*P*_1_. Discrepancy between the two discretizations occurs because the flow is counted twice as it is fed from one voxel to the other. Right: As a solution to the described problem we rather pick out a true distribution volume Δ*V* (area in this 2D sketch), which is a small area around a given streamline along the centre line of the grey area. This is the true distribution volume (area in this 2D sketch) which is fed with arterial blood from the incoming fractional flow Δ*F*_0_. The correct perfusion within Δ*V* is therefore Δ*F*_0_/Δ*V*. The entire compartment can further be divided into similar infinitesimal distribution volumes, thus providing locally correct perfusion estimates.

On the other hand, if the control region is a single voxel or a sub-division of a capillary system with sequentially feeding arterial blood, the traditional model assumptions are violated since every control region will feed its neighbours, thus becoming a coupled system of flow. Simply summing the total inflow into a voxel and dividing by the voxel volume will overestimate the perfusion as the normalization refers to the wrong volume. This phenomenon is demonstrated in Fig 2 where the volume on the left has the true perfusion of *P*_1_ = *F*_0_/(2*V*) for an incoming flow *F*_0_ [mm^3^s^−1^] and distribution volume 2*V* [mm^3^]. However, for another discretization shown in the middle, the perfusion within each of these sub-volumes becomes *P*_2_ = *F*_0_/*V* = 2*P*_1_. Taking the average across the two sub-volumes, it is clear that the perfusion is overestimated with a factor of two. A discretization dependent perfusion estimate is not recommendable, and the perfusion estimate of *P*_2_ is clearly wrong.

In the following we introduce a meaningful notion of perfusion for the fine scale continuous model. To do this, we will consider distribution volumes which are following the streamlines, as shown in Fig 2 (right). For each point of a streamline we will select a small perpendicular disk with radius chosen in such a way that the total flow over each disk is constant along the streamline.

More precisely, let us consider an arbitrary streamline $S\subseteq \Omega \subseteq R^{3}$ of length *l* > 0 and parametrization *s*: [0, *l*] → *S*. We start by calculating the total flow over a small 2D disc perpendicular to the streamline. Let **y** ∈ *S* be an arbitrary location along the streamline. The total flow *F* over a 2D disc *B*(**y**, *R*(**y**)) perpendicular to the flow field **q**(**y**), centered at **y** and with radius $R:S\to R^{+}$, is given by

$$\begin{array}{c} F\left(y,R\left(y\right)\right)=\int_{B(y,R(y))}q\left(x\right)\cdot ndx\;\text{where}\;n:=q\left(y\right)/\left|q\left(y\right)\right|. \end{array}$$ (13)

In order to calculate the perfusion, we need to establish the volume of a small tube around the streamline. We will not consider a tube with constant radius, but one with spatially varying radii $r:\left[0,l\right]\to R^{+}$. The total volume of such a tube is given by

$$\begin{array}{c} V\left(r\right)=\int_{0}^{l}r{\left(u\right)}^{2}\pi du. \end{array}$$ (14)

Note that *R*(**y**) ≔ *r*(*u*) for some *u* ∈ [0, *l*]. We define the perfusion at the arbitrary point **y** on the streamline as

$$\begin{array}{c} P_{s}\left(y\right):=\lim_{\varepsilon \to 0}\frac{F(y,\varepsilon R(y))}{V(\varepsilon r)}\;\text{for}\;R\left(y\right):=1/\sqrt{\left|q(y)\right|}. \end{array}$$ (15)

In this expression the radii *R*(**y**) are chosen in such a way that in the limit when *ε* → 0, the perfusion is constant along the streamline. To see this, let us assume that **q** is differentiable with Jacobian *J*. Using a Taylor expansion of **q**(**x**) around **y**, the Lagrange remainder theorem, as well as a change of coordinates **z** = (**x** − **y**)/(*εR*) yields

$$\begin{array}{c} F\left(y,\varepsilon R\left(y\right)\right)=\varepsilon^{2}\left(\pi +\varepsilon \int_{B(0,1)}n^{⊤}J\left(\zeta \right)zR{\left(y\right)}^{3}dz\right) \end{array}$$ (16)

where *ζ*_*i*_ ∈ (0, *z*_*i*_) for every vector element *i*, and simplifications are due to $R\left(y\right)=1/\sqrt{\left|q(y\right)}|$ and **n** ≔ **q**(**y**)/|**q**(**y**)|. Combining this result with (15) yields what we refer to as global perfusion

$$\begin{array}{c} P_{s}\left(y\right)={\left(\int_{0}^{l}r{\left(u\right)}^{2}du\right)}^{-1}. \end{array}$$ (17)

Note that (17) is independent of the spatial location **y** along the streamline, and is an explicit formula for converting flux into perfusion, showing that the perfusion scales with the streamline length *l*, as well as with the geometry of the domain, represented by the radii *r*(*u*).

### 2.6 A method to estimate local porosity

Porosity and CBV have the same definition, and we can therefore state that *ϕ* ≡ CBV. It is known from literature on traditional models [1] for perfusion that CBV for the entire compartment can be expressed as

$$\begin{array}{c} \phi =\frac{\int_{0}^{\infty }C\left(s\right)ds}{\int_{0}^{\infty }c_{\text{a}}\left(s\right)ds}. \end{array}$$ (18)

It is not obvious that (18) is valid also for a 1C field model where the voxels are feeding each other. We will now show that this is indeed the case.

Let us switch to a discrete setting. Consistent with the considerations in Section 2.4, the CA concentration in any voxel can be described by *C*_*i*_(*t*) = *ϕ*_*i*_(*J*_*i*_ ∗ *c*_in,*i*_)(*t*), where the local arterial input is given by *c*_in,*i*_(*t*) = 1/*P*(*P*_0_*c*_a_(*t*) + ∑_*j*∈*J*_*P*_*j*_*c*_*j*_(*t*)) and *J*_*i*_(*t*) = (*P*/*ϕ*)*e*^−(*P*/*ϕ*)*t*^. In *c*_in,*i*_(*t*), *J* is the index set of all adjacent, upstream voxels and *P* = *P*_0_+∑_*j*∈*J*_*P*_*j*_. Here *P*_*j*_ is the normalized volume flow across voxel-face *j* [mm^3^s^−1^mm^−3^] and *P*_0_ > 0 if voxel *i* has arterial contribution. Furthermore, let us assume that **q** is a uni-directional flow field across each voxel face.

We will now use induction to show that $\int_{0}^{\infty }c_{i}\left(s\right)ds=\int_{0}^{\infty }c_{\text{a}}\left(s\right)ds$, and then (18) follows. Let *I*_*k*_ denote the set of voxels which have *k* layers of upstream voxels. E.g. *I*_0_ is the set of all voxels, which have no upstream voxels, *I*_1_ is the set of voxels which are fed by *I*_0_ and so on. As an assumption, the same voxel can not be member of several *I*_*k*_, thus there is no flow interaction between voxels within the same *I*_*k*_. Induction will be carried out over *k*.

**Induction basis:** Let *k* = 0 and let *i* ∈ *I*_0_ be arbitrary. Since the area under the convolution of two functions equals the product of the area of its factors, $\int_{0}^{\infty }c_{i}\left(s\right)ds=\int_{0}^{\infty }c_{\text{a}}\left(s\right)ds$ and the claim follows.

**Induction step:** Consistent with our assumptions, for any voxel at location *i* ∈ *I*_*k*+1_ which has the voxels *J* ⊆ *I*_*k*_ as their upstream neighbors, we find the following expression:

$$\begin{array}{c} \int_{0}^{\infty }c_{i}\left(s\right)ds=\frac{1}{P}\int_{0}^{\infty }\left(J_{i}*\left(P_{0}c_{\text{a}}\left(s\right)+\sum_{j\in J}P_{j}c_{j}\left(s\right)\right)\right)\left(s\right)ds \end{array}$$ (19)

Splitting the convolution integrals into separate factors, applying $\int_{0}^{\infty }J_{i}\left(s\right)ds=1$, as well as the definition of *P* yields the claim.

### 2.7 Simulation of capillary flow

In this section we describe how we simulate a flux field **q**(**x**) driving the transport of fluid and tracer. The modeling is in agreement with previous work on capillary perfusion simulations [14–16].

For the time being we will not consider contrast agent concentrations, but only the fluid flow in general. In-line with standard theory for a steady-state flow of an incompressible fluid and with Darcy’s law [17], we assume that the fluid-flow **q**(**x**) obeys the following set of local PDEs

$$\begin{array}{c} \nabla \cdot q=Q,\;\text{where}\;q=-\frac{k}{\mu }\nabla p. \end{array}$$ (20)

Here *Q* [mm^3^s^−1^mm^−3^] is the user-defined source- and sink term, which we assume to be only non-zero within the source or the sink, **k** = **k**(**x**) is the intrinsic permeability tensor, *p*(**x**) is the pressure, and *μ*(**x**) is the viscosity of the fluid. For simplicity, we will assume that **k** is a symmetric and positive definite tensor with only nonzero diagonal elements **k**_*ii*_ = *k*, in accordance with a homogeneous porous medium. Using (20) yields the following elliptic partial differential equation in the pressure field *p* within the closed domain Ω,

$$\begin{array}{c} |\begin{array}{cc} \nabla \cdot (-\frac{k}{\mu }\nabla p)=Q, & x\in \Omega ,\\ q\cdot n=0,\;\; & x\in \partial \Omega , \end{array}| \end{array}$$ (21)

for the outward unit normal **n**(**x**). After solving (21) for the pressure *p*, the flux field was estimated according to (20).

### 2.8 Numerical implementation

First solving (21), and then (8), we set up a forward simulation of blood flow and indicator dilution through the capillary system. A standard arterial input function was chosen [9], the gamma-variate function *c*_a_(*t*) ≔ *D*_0_(*t* − *t*_0_)^*α*^ *e*^−(*t* − *t*_0_)/*β*^ for default parameters *α* = 3, *D*_0_ = 1 mmol l^−1^s^−1^, *β* = 1.5 s and *t*_0_ = 0 s. Average, ground truth perfusion was chosen as 50 ml/min/100ml, a typical value for human brain perfusion [18, 19]. The field of view was chosen as 2mm × 2mm × 2mm, in the order of the capillary bed or individual capillaries, ranging from 0.1 mm to 3 mm [14], or 0.25 mm to 0.850 mm [20]. The source term was assigned to the upper left voxel and the sink term was assigned to the lower right voxel. The source can be understood as the arterial compartment, the sink as the venous compartment, and the remaining field of view as the capillary system. The arterial input function (AIF) was measured in the source. Permeability was chosen to be isotropic and constant throughout the domain **k** = *k***I** for the identity **I** and *k* = 5 × 10^−6^ mm^2^. Dynamic blood viscosity was chosen as *μ* = 5 × 10^−6^ kPas according to [21]. Porosity (e.g. CBV) was assumed to be *ϕ* = 0.05, in line with measured CBV of the brain [19].

Eq (21) was solved numerically using two-point flux-approximation (TPFA), well known within porous media simulations [22]. The transport of CA described in (7) was implemented using first order upwinding [23], yielding a discrete 2D+time CA concentration map *C*(**x**_*i*_, *t*_*j*_). From the porous media model using (21) and (8), streamlines to compute global perfusion *P*_*s*_ were found from tracking of the flux vector field **q** by FACT [24], known from tractography.

Prior to reconstruction of perfusion using traditional models, the CA concentration map *C*(**x**_*i*_, *t*_*j*_) was downsampled to a time-resolution of .1s. In order to simulate different spatial resolutions of the scanning process, the data was averaged into blocks of {1, 2, 4, 8, 16, 32, 64} voxels with corresponding voxel sizes. Success of restoration was measured in terms of absolute, relative reconstruction error, RE(*a*, *b*) ≔ 100% ⋅ |*a* − *b*|/*b*, where *a* is reconstructed values and *b* is ground truth values. Local perfusion *P*_v_ was computed according to (10). Global perfusion *P*_s_ was computed according to the streamline definition (17).

### 2.9 Reconstruction of perfusion within real data

In order to illustrate the effect of overestimation also in real data we applied the deconvolution model to a clinically acquired human perfusion CT dataset of a 56 years old male admitted with suspicion of stroke to the Radboud University Medical Center in Nijmegen, the Netherlands. The perfusion scan was obtained using a Toshiba Aquilon ONE scanner, voxel-size 0.43 mm × 0.43 mm, slice thickness 0.5 mm, contrast agent 50 ml Xentix 300, total scan-time 114 s, time resolution ranging from 2.1 s in the early- to 30 s in the late phase of CA uptake. Motion correction was performed with respect to the first timepoint using Euler transformations [25]. The arterial input function was manually selected within the middle cerebral artery (MCA) by a medical expert. Since we expected to see local overestimation effects mainly for small voxel sizes, the data was processed at full resolution (512 × 512 × 320 voxels). To cope with noise, we applied gaussian smoothing with standard deviation of 1 voxel and window size [5, 5, 5]. Relative concentrations were estimated from the CT signal assuming a spatially independent proportionality constant. The brain tissue was segmented automatically and used as ROI for the perfusion analysis.

## 3 Results

### 3.1 Reconstruction of perfusion within synthetic data

**Fig 3:**
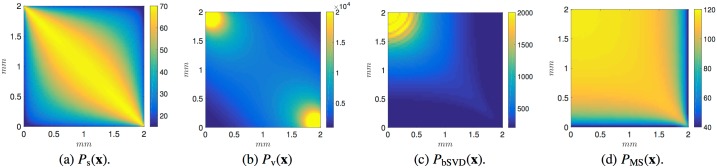
Ground truth and reconstructed perfusion maps. Ground truth (a-b) and reconstructed (c-d) perfusion maps [ml/min/100ml] at the lowest discretization scale. The reconstructed perfusion maps have substantially varying characteristics compared to any of the two grond truth perfusion maps. (a) Global perfusion *P*_s_(**x**) along the streamlines according to (17). (c) Local perfusion *P*_v_(**x**) according to (10). (c) Reconstructed perfusion *P*_bSVD_ according to (3). (d) Reconstructed perfusion *P*_MS_ according to (5).

Tracer dilution in the flux-field was simulated and from the resulting intensity time curves we tested the convolution based traditional model (bSVD) (3) as well as the maximum-slope (MS) model (5) for their capability to recover perfusion. Recovered perfusion maps *P*_bSVD_ and *P*_MS_ were compared against the two ground truth perfusion maps *P*_s_ and *P*_v_ depicted in Fig 3. As an internal control of *P*_s_, the average *P*_s_ at maximal resolution was found to be 49.59 ml/min/100ml, for all practical means identical to the global input perfusion of 50 ml/min/100ml mediated through the source. Results from reconstruction of the porosity *ϕ* (i.e. CBV) according to (18) resulted in reconstruction errors of <1% for all voxel sizes.

**Fig 4:**
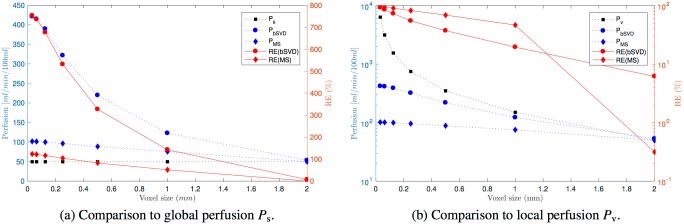
Restored perfusion as a function of voxel size. Comparison of restored perfusion with ground truth perfusion as a function of varying voxel size. Dotted, blue lines show average perfusion (left axis). Solid, red lines are average, relative errors (RE) of restored perfusion as compared to ground truth perfusion (right axis). (a) Global perfusion *P*_s_ is independent of discretization. Subdivision of the domain into smaller cells leads to a substantial overestimation of perfusion for both reconstruction methods. (b) Local perfusion *P*_v_ is dependent on discretization level. A subdivision of the domain leads to substantial underestimation of perfusion when compared to *P*_v_ for both reconstruction methods.

We performed two different normalizations of the restored flow, a normalization (i) with respect to volume, and (ii) with respect to surface. The volume normalization (i) implies normalizing the flow to [ml/min/100ml], most in line with common units for perfusion. A comparison of ground truth perfusion to reconstructed perfusion using volume normalization is shown in Fig 4. For a voxel size corresponding to the entire ROI (voxel size = 3 mm) the reconstructed perfusion of *P*_bSVD_ and *P*_MS_ is close to the ground truth perfusion *P*_s_ and *P*_v_. For any voxel size smaller than the entire domain the relative error increases inversely with voxel size, in particular for reconstruction by bSVD. Global perfusion *P*_s_ is not depending on voxel size.

**Fig 5:**
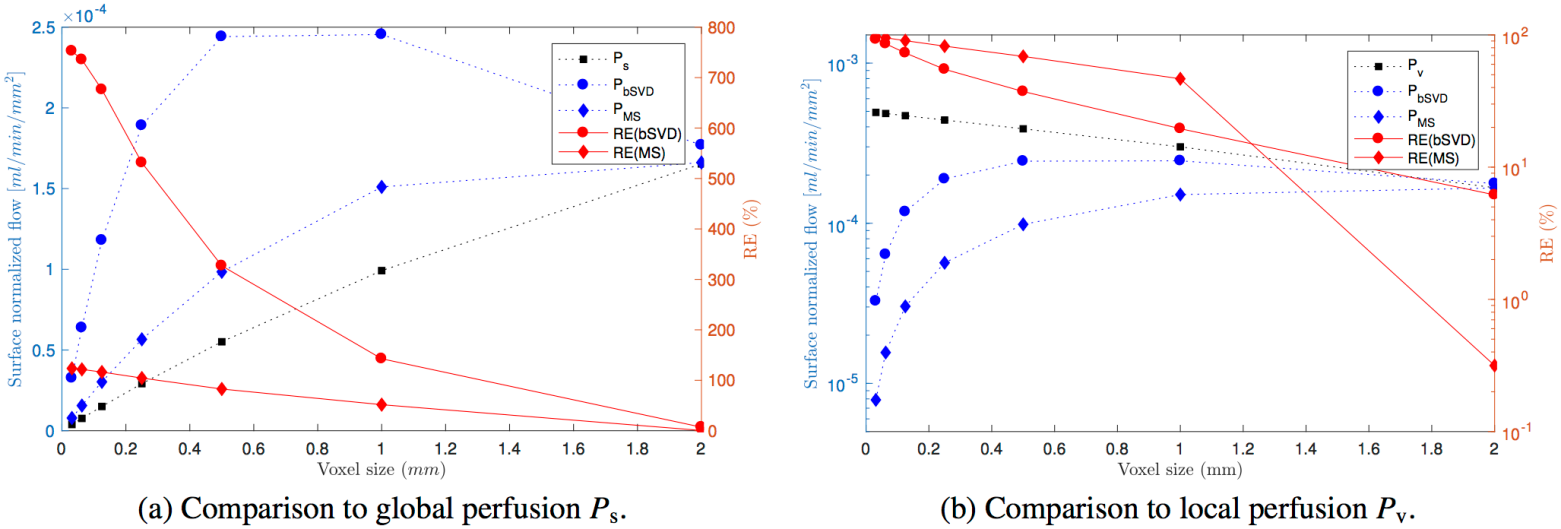
Surface normalized flow. Comparison of surface normalized reconstructed flow [ml/min/mm^2^] to the ground truth as a function of varying voxel size. Dotted, blue lines show average surface normalized flow (left axis). Solid, red lines are average, relative errors as compared to flow estimated from ground truth perfusion (right axis). (a) Black, dotted line with filled squares shows that surface normalized flow estimated from ground truth perfusion *P*_s_ is dependent on discretization level. Subdivision of the domain into smaller voxels leads to substantial overestimation of surface normalized flow for both reconstruction methods bSVD and MS. (b) Black, dotted line with filled squares shows that surface normalized flow estimated from local perfusion *P*_v_ is also dependent on discretization level. A further subdivision of the domain leads to substantial underestimation of the flow for both reconstruction methods bSVD and MS.

For surface normalization (ii) we first computed the absolute flow *F* [ml s^−1^] of ground truth perfusion as well as reconstructed perfusion, and then normalized the flow to the surface area of the distribution volume, *F*/*S*, here referred to as surface normalized flow. This interpretation of perfusion has no standard clinical unit, thus we choose to scale the surface area *S* to [mm^2^]. Reconstruction results of surface normalized flow is shown in Fig 5. For a voxel size corresponding to the entire ROI (voxel size = 3 mm isotropic) the surface normalized flow of *P*_bSVD_ and *P*_MS_ is close to the ground truth. For any voxel size smaller than the entire domain the relative error increases inversely with voxel size, in particular for reconstruction by bSVD. Both global perfusion *P*_s_ and local perfusion *P*_v_ are dependent on voxel size in the framework of surface normalized flow.

The difference in nature of surface vs. volume normalized flow makes a direct comparison of flow values in Figs 4 and 5 cumbersome. In addition, the absolute values deviate in several orders of magnitude due to different scale of the normalization factors, [100ml] vs. [mm^2^], respectively.

### 3.2 Reconstruction of perfusion within real data

**Fig 6:**
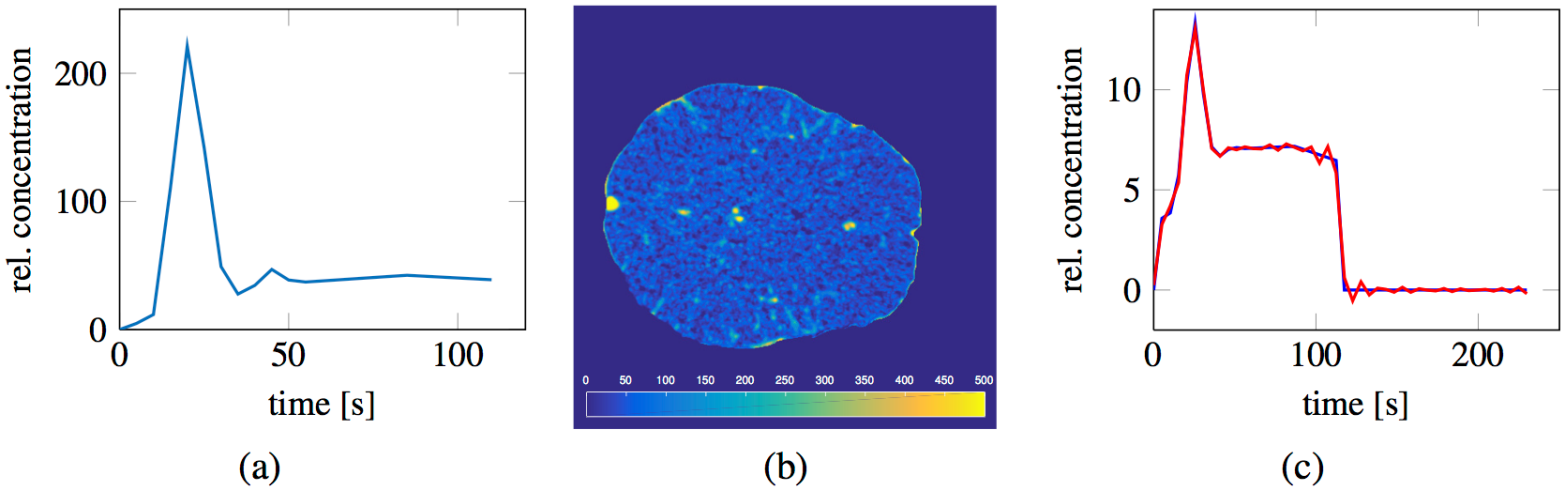
Reconstructed perfusion in real data. Real-data reconstruction of perfusion (see Section 2.9 for details). (a) AIF manually selected from the MCA. (b) One slice of restored voxelwise CBF [ml/min/100ml] from a 3D volume of interest. (c) Mean concentration time curve for the complete 3D volume of interest and the curve approximation by bSVD (rel. = relative).

Perfusion for the entire brain by averaging the concentration time curves first and then performing the bSVD yielded a perfusion of *P*_bSVD_ = 24.79 ml/min/100ml. As a second step, voxelwise perfusion was estimated, depicted in Fig 6. These values yielded an average perfusion of ${\bar{P}}_{\text{bSVD}}=$ 64.36 ml/min/100ml, corresponding to an overestimation of perfusion with *RE* = 159.60% compared to the value obtained for the entire brain.

## 4 Discussion

It has previously been shown that perfusion reconstructed from traditional 1C models in a coupled system is discretization dependent (cfr. Fig 2) [6–8]. As a consequence, the obtained results will strongly depend on acquisitions parameters and post-processing tools. It is unknown to which extent the pharmacokinetic modelling overestimates perfusion and whether the error is homogeneously distributed or not. Considering this, the shortcoming of existing perfusion formulations has not been sufficiently well accounted for within clinical studies [26, 27]. To clarify the potential impact of limitations seen within existing perfusion models, our main contribution in the current work is to quantify the observed error. To the best of our knowledge, such quantification has not been carried out previously.

Our results strongly support the usage of traditional 1C models for entire regions exclusively fed by the measured arterial input. Moreover, our results also show that when traditional models are applied only to parts of the system, the measured perfusion is overestimated (cfr. Fig 4, black and blue curves). Observed error in perfusion for a voxel size of ∼2 mm was found to be ∼ 40% for reconstruction by bSVD and ∼ 20% for reconstruction by MS (cfr. Fig 4). This is a relevant spatial scale for today’s MR acquisitions. The error is expected to increase in future acquisitions along with hardware and software improvements leading to higher spatial sampling.

There are at least two reasons for overestimation of perfusion in traditional 1C models. The first reason is that blood passing through a voxel without being locally delivered to the capillary tissue will contribute to artificially high perfusion values. This issue has not been accounted for in our digital model but my be handled by more complex multi compartment spatial modelling described in i.e. [8]. The second reason is thoroughly described here, and relates to estimation of an incorrect distribution volume used for computing the perfusion. Overestimation of perfusion obtained within the digital phantom was also confirmed by real data experiments, where we showed local overestimation of perfusion for voxelwise estimates as compared to an averaging of concentrations for the entire volume of interest (cfr. Section 3.2).

In order to demonstrate our results we introduced two definitions of voxelwise perfusion, global perfusion *P*_s_ and local perfusion *P*_v_. Local perfusion *P*_v_ is in line with [7] where the authors demonstrated a discretization dependent flow without connecting it mathematically to perfusion. Theory and examples in our work show that this definition of perfusion does not comply with the physical understanding of perfusion as a feeding arterial blood flow. The correct distribution volume is not accounted for and the obtained perfusion will be strongly overestimated compared to the actual perfusion. However, our analyses show that traditional models would restore the local flow value if the local arterial input function was selected, implying that traditional models are accurate as long as the model assumptions are not violated. The coupling between the continuous porous media model and the convolution model in Section 2.4 demonstrates that there is no contradiction between these two models. The problematic issue of traditional models is related to physical interpretation and normalization with respect to incorrect distribution volume.

Global perfusion *P*_s_ models perfusion along the streamlines and most accurately reflects the physical notion of volume flow within the correct distribution volume according to mathematical definitions. We showed that *P*_s_ is independent of discretization (cfr. Fig 4), *P*_s_ is a constant quantity along the streamline, and scales with streamline length and geometry according to (17).

For our purpose, the concept of *P*_s_ was useful as a realistic ground truth in order to clarify the definition of perfusion as a flow that must be normalized along the entire capillary length, where the blood undergoes a transition from arterial to venous blood. Traditional perfusion measurements are thereby dependent on both the discretization and the geometry of the domain. While the geometry in our experiments is limited to a simplified square with diagonal flow gives fairly simple analysis, estimation of *P*_s_ in real applications is practically difficult due to a highly complex and unknown microvasculature. Nevertheless, the derivation of the theoretical relation between *P*_s_ and streamline length is valid for an arbitrary geometry.

Development of new field models for perfusion is highly demanded due to the scale dependency, and a few initiatives have been proposed based on multi compartment flow models [8, 16]. Alternative approaches with estimation of voxel-speciffic AIFs have also been suggested [28, 29]. These approaches may suffer from instabilities or errors which have not yet been handled [29]. A possible hybrid approach that may have clinical relevance is to estimate a modest number of local AIFs feeding anatomically sensible regions on a scale way above the capillary systems. This will however require some prior knowledge of local anatomy and vasculature.

It was previously suggested to normalize the flow by surface instead of volume [7]. Our experiments suggest that a surface normalization is nevertheless discretization dependent, and traditional 1C models are not able to restore this type of perfusion, neither for global, nor for local perfusion (cfr. Fig 5, blue and black lines).

We have also shown there is a global error directly scaling with smaller voxel sizes (cfr. Fig 4). A comparison between individual scans with otherwise equal acquisition parameters and post-processing chains should ideally adjust for the global error in the interpretation of absolute perfusion values. As such, voxelwise maps of perfusion could still be of high clinical value as the main goal is a comparison of perfusion between patients or between repeated scans of individuals. However, particular care should be undertaken in the case of comparing perfusion data of various resolution. Multicenter or retrospective studies are particularly susceptible to this issue where data collected from various sources are different with respect to hardware, resolution and post-processing tools that can affect the discretization level. Future study design should account for this limitation and special care should be undertaken to ensure equal level of discretization in perfusion estimates.

In addition to the observed global error we also observed inhomogeneous reconstruction errors within the capillary system. This becomes clear in Fig 3 where the reconstructed perfusion maps *P*_bSVD_ and *P*_MS_ are strongly unlike the ground truth perfusion maps of *P*_s_ and *P*_v_. This inhomogeneity leads to locally inaccurate estimates of perfusion within a patch, even if the global average value within the patch were correct. If analyses of voxelwise perfusion is undertaken with high resolution the local error can become large within each capillary patch.

Regarding the CBV estimates, estimation of blood volume is stable, and varying voxel size had little impact on the results. These results are in agreement with the analyses in Section 2.6 supporting the usage of (18) for computing voxelwise CBV with high accuracy for any voxel size.

## 5 Conclusion

Our experiments confirm that traditional 1C models for perfusion perform well if they are applied to the entire domain. However, when they are applied to fractions within a coupled domain, perfusion becomes scale dependent. We quantified substantial and increasing reconstruction errors of perfusion as a function of smaller voxel size, and we also found similar effects in real data. The observed reconstruction error for a simplified geometry in clinically relevant resolution was between ∼20% to ∼40%. The error is expected to vary with geometrical complexity of the capillary system and increase with higher spatial resolution. The reason for the observed errors is not numerical instabilities in the deconvolution but rather that traditional 1C models will not account for correct distribution volume within smaller fractions of the region of interest. As a consequence, interpretation of absolute perfusion for the purpose of diagnosis and disease monitoring must be undertaken with care, and comparison of perfusion values from individual dynamic data sets with different resolution is not recommended.
